# Late-age onset systemic sclerosis—clinical and serological characteristics

**DOI:** 10.1007/s10067-024-07025-z

**Published:** 2024-06-21

**Authors:** Ewa Wielosz, Katarzyna Wiąk-Walerowicz, Ewa Łyś, Aleksandra Lipska, Magdalena Dryglewska, Maria Majdan

**Affiliations:** Department of Rheumatology and Connective Tissue Diseases, Medical University of Lublin, Jaczewskiego 8 St., 20-090 Lublin, Poland

**Keywords:** Elderly, Geriatrics, Late onset, Systemic sclerosis

## Abstract

The clinical course and serological profile of the late-age onset systemic sclerosis (LAO SSc) and the early-age onset SSc (EAO SSc) was compared. The study enrolled 157 patients that fulfilled the American College of Rheumatology (ACR)/European League against Rheumatism (EULAR) classification criteria for systemic sclerosis (SSc). Among them, 69 had diffuse cutaneous SSc (dcSSc) and 88 limited cutaneous SSc (lcSSc). Within this population, 39 patients developed the disease over the age of 60 years old (LAO SSc) and 118 prior to that age (EAO SSc). The subtype of SSc, the incidence of internal organ involvement, the prevalence of malignancy, mortality, and serological profile were compared between both groups. The LAO SSc was observed in 39 of total 157 patients with SSc and exhibited a notably higher prevalence of pulmonary arterial hypertension (*p* = 0.014), heart involvement (*p* = 0.0014), and renal involvement (*p* = 0.0002). The occurrence of arthralgias was less common in the LAO SSc group (*p* = 0.02) than in the EAO SSc group. Furthermore, in the LAO SSc group, the prevalence of anti –RNA polymerase III antibodies (*p* = 0.008) and antiPM/Scl antibodies (*p* = 0.048) were significantly lower than in the EAO SSc group. On the other hand, higher anti-Th/To antibody levels (*p* = 0.014) were recorded in the LAO SSc group. Approximately 25% of SSc patients experienced a delayed onset of the disease after the age of 60 years old. Some clinical and serological features of late-onset SSc were markedly different from that in early-onset disease. Particularly noteworthy is the fact that involvement of internal organs such as heart and kidneys, as well as pulmonary arterial hypertension were much more often observed among patients with LAO SSc which in our suggestion may be referred to age-related co-morbidities.

**Table:** 

**Key Points** *• Significant differences in clinical and serological profile of the disease were found between late-age onset (LAO) and early-age onset (EAO) SSc.* *• Incidence of dcSSc as well as prevalence of anti–RNA polymerase III and anti-PM/Scl antibodies were found to be lower in patients over 60 years old compared to those before 60, but regardless of the age of the disease onset.* *• Internal organ morbidity, notably pulmonary arterial hypertension, renal impairment and heart disease were significantly more common in elder SSc patients as well as in those with late disease onset.* *• These findings may suggest an impact of age-related co-morbidities on the course of late-age onset SSc.*

**Key Points**

*• Significant differences in clinical and serological profile of the disease were found between late-age onset (LAO) and early-age onset (EAO) SSc.*

*• Incidence of dcSSc as well as prevalence of anti–RNA polymerase III and anti-PM/Scl antibodies were found to be lower in patients over 60 years old compared to those before 60, but regardless of the age of the disease onset.*

*• Internal organ morbidity, notably pulmonary arterial hypertension, renal impairment and heart disease were significantly more common in elder SSc patients as well as in those with late disease onset.*

*• These findings may suggest an impact of age-related co-morbidities on the course of late-age onset SSc.*

## Introduction

Systemic sclerosis (SSc) is a multi-organ connective tissue disease that involves dysfunction and impaired morphology of the blood vessels, non-specific inflammation, and progressive fibrosis. A diagnostic work-up used for early diagnosis of SSc includes serum antinuclear autoantibodies (ANAs) and nailfold capillaroscopy (NVC)—a simple and non-invasive imaging tool that allows visualization of skin microcirculation. A specific NVC pattern (scleroderma pattern) is considered a marker of the disease and its recognition has recently been improved by digital imaging systems and validated programs for automated NVC image evaluation [1, 2]. The clinical course of the disease is driven by a number of factors including SSc subtype, organ dysfunction and damage, serological profile, comorbidities, gender, race, and age. Everyday activities, ability to work, and patients’ self-existence are often limited by the disease. Thus, such patients require multidisciplinary medical care with regular assessment and effective treatment [3].

Although typically, SSc affects patients between 30 and 50 years of age, the cases of disease onset before 20 years of age, after 60–70 or even 75 years of age have also been reported. According to literature data, approximately 10–20% of SSc cases are classified as late-onset, i.e., diagnosed after 60–65 years of age [4–6]. Moreover, in a North American cohort study, White patients were diagnosed with SSc at an older age compared to black ones [7]. The late-age onset (LAO) SSc seems to be distinctly different from that of the early-age onset (EAO). In terms of clinical presentation, it is the course of the disease and the ANAs profile [8, 9]. Controversies still exist on the associated mortality rate in relation to age in SSc, with some reports evidencing that LAO SSc is associated with poorer prognosis and lower survival rates [5, 10]. On the other hand, according to previous studies, LAO SSc was characterized by a milder course with limited morbidity, minimal skin involvement, and limited organ damage [11–13]. In fact, the notion that limited cutaneous SSc is more commonly observed in late-onset patients is shared by the majority of studies; however, there are also opposite data from a Hungarian and an American cohort demonstrating that the majority of patients with LAO SSc had diffuse disease subtype [14–16]. It is also unclear whether the frequency of organ involvement correlates with LAO SSc. In addition, algorithms for diagnosing organ involvement in SSc are still being developed, e.g., there are several criteria of varying specificity for the diagnosis of pulmonary arterial hypertension [17]. Thus, determining the pattern of the development and course of a disease that is characterized by multiple organ complications such as SS seems to be of special importance in an elderly patients’ population due to the existence of age-related co-morbidities and pharmacotherapy used, the insight in this matter may also be noteworthy in view of new management and diagnostic algorithms. In this regard, this study was set out primarily to compare the serological status and clinical course of the disease in LAO SSc and EAO SSc patients with particular respect to the internal organs involvement, including heart, lungs, kidneys, the gastrointestinal tract, and the musculoskeletal system. An additional aim was to assessed whether clinical and serological features differ according to patient age. We also are taking into considerations that differences could be related to age-related comorbidities.

## Patients and methods

The study population consisted of 157 European Caucasian patients with SSc (119—females and 38—males), admitted to the Department of Rheumatology and Connective Tissue Diseases of the Medical University of Lublin, Poland. The patients fulfilled the current (2017) ACR/EULAR SSc classification criteria [18]. Before enrolment, patients gave their written informed consent for participation in the study. The disease duration was determined and patients were categorized according to the Le Roy et al. classification criteria as having limited cutaneous SSc (lcSSc) or diffuse cutaneous SSc (dcSSc) [19] (Table 1). Clinical and serological features of SSc were established in each patient. The cohort was divided into two groups according to patient’s age at disease onset—group I—patients diagnosed after the age 60 years, i.e., LAO SSc—39 patients; and group II—patients diagnosed before the age 60 years, i.e., EAO SSc—118 patients. Additionally, regardless of the age of the disease onset, all patients were divided into two new groups: group A—those before 60 y.o.—82 patients, and group B—those over 60 y.o.—75 patients. The groups were subsequently compared and contrasted with respect to SSc subtype, the incidence of internal organ involvement, the prevalence of malignancy, mortality rate, and serological profile. The organ damage was assessed based on clinical symptoms, imaging and laboratory diagnostics. On lungs’ high-resolution computed tomography (HRCT), two types of interstitial lung involvement were considered—ground-glass opacities and bibasilar fibrosis. The pulmonary function was assessed using carbon monoxide diffusion capacity (DLCO) and total lung capacity (TLC) parameter on spirometry. Heart involvement was established as the presence of arrhythmia, conduction disturbances, or heart failure. Pulmonary artery systolic pressure (sPAP) over 35 mmHg as measured at rest by Doppler echocardiography was indicative of pulmonary arterial hypertension (PAH). Myalgia or myositis were considered as pain or muscle weakness, and/or elevated serum phosphocreatine kinase (CPK) levels. Joint involvement was diagnosed based on joint tenderness (arthralgia) or tenderness and swelling (arthritis). The gastrointestinal (GI) tract was evaluated based on a barium swallow test, and symptoms such as dysphagia, heartburn, diarrhoea, and bloating were reported. The signs of renal disease were the presence of scleroderma renal crisis (SRC)or abnormal renal function tests such as decreased eGFR, proteinuria, or high serum creatinine level. Calcinosis, digital ulcerations, and contractures were also assessed. Additionally, the groups were analysed comparatively with regards to the presence of overlapping autoimmune thyroid diseases (ATDs). The presence of ANAs in patients’ sera was detected using EUROLINE Systemic Sclerosis Profile (EUROIMMUN AG, Lubeck, Germany) determining antibodies against SSc-specific antigens: anti-Scl70, anti-centromere (ACA), anti-RNA polymerase III, anti-PM/Scl, anti-fibrillarin, anti-Ku, anti-Th/To anti-Ro52, anti-PDGFR (platelet-derived growth factor receptor), and anti-NOR90 antibodies. Procedure was performed according to the manufacturer’s protocol. The data were acquired and interpreted using EUROLINE Scan (EUROIMMUN AG, Lubeck, Germany).

**Table 1:** Clinical characteristics of systemic sclerosis patients

Number of patients	157
Gender	
Female	119
Male	38
Age (years)	58.1 (23–81)
Subtype of SSc	
dcSSc	69
lcSSc	88
Duration of disease (years)	5.15 (0.0–23)

The statistical analysis was carried out using STATISTICA v. 13.0 software (StatSoft, Cracow, Poland) using the non-parametric Chi-square test. A *p* value < 0.05 was considered significant. Analyzed data were expressed as number (percentage) for categorical variables and mean (SD) or range (minimum–maximum) for continuous variables. Statistical significance of differences in the frequency of clinical and laboratory symptoms characteristic of SSc in age-dependent patient groups was examined using the exact Pearson’s and Fisher’s Chi-squared test and the test of the significance of differences for two structure indicators. Two-sided *p* values less than 0.05 were considered statistically significant.

## Results

Based on the collected data, LAO SSc group included 39 out of 157 patients with SSc (approximately 25%). This group displayed a substantially higher prevalence of pulmonary arterial hypertension (*p* = 0.014), heart and renal involvement (*p* = 0.0014 and *p* = 0.0002, respectively), whereas arthralgias were less common than in the EAO SSc group (*p* = 0.02). Other clinical manifestations, such as interstitial lung disease, GI tract involvement, calcinosis, digital contractures, myalgia, SRC, arthritis, neoplastic diseases, mortality rate, and overlapping ATDs, were observed with a similar frequency in both of those SSc groups (Table 2). Detailed comparative data is given in Table 2. Considering the ANAs profile, in the LAO SSc group, the prevalence of anti-RNA polymerase III and anti-PM/Scl antibodies was significantly lower than in the EAO SSc group (*p* = 0.008 and *p* = 0.048, respectively), as opposed to anti-Th/To antibodies that were significantly more common in LAO SSc (*p* = 0.014). In addition, a tendency to a higher prevalence of anti-centromere antibodies in the LAO SSc group was observed, however without statistical significance (*p* = 0.07) probably due to a small sample size (Table 3). Moreover, when comparing group A (patients before 60 y.o.) and group B (patients over 60 y.o.), dcSSc subtype was less frequent in the latter, regardless of the age of disease onset (*p* = 0.03) (Table 4). In addition, the frequency of both GI tract and renal involvement were notably higher in group B than group A (*p* = 0.04 and *p* = 0.0014, respectively). Similar tendency was observed with respect to pulmonary arterial hypertension, which was more common in patients after the age of 60 years (25.3% vs. 14.6%), however without significant statistical confirmation. Otherwise, mortality rates exhibited higher values in patients < 60 y.o. when compared to elder group (19.5% vs. 10.7%). In terms of other investigated clinical symptoms: interstitial lung disease, calcinosis, digital contractures, myalgia, SRC, arthralgias, arthritis, neoplastic diseases, or co-existence of ATDs, no relevant differences were found between groups A and B (Table 5). The serological profiles were comparable in those groups, except of the higher prevalence of anti-Scl-70 antibodies in the group of SSc patients before the age 60 years (Table 6).

**Table 2:** Comparison of the clinical parameters in group I (late-age onset—after age 60 years) and group II (early-age onset before age 60 years**)** patients with systemic sclerosis

	Group I late-age onset *n* = b39	Group II early-age onset *n* = 118	*p*
ILD (HRCT)	20 (51.3%)	64 (54.2%)	NS
Decreased DLCO	19 (48.7%)	48 (40.7%)	NS
Heart involvement	24 (61%)	38 (32.2%)	0.0014
PAH (ECHO)	13 (33.3%)	18 (15.25%)	0.0141
Myalgia or myositis	6 (13.0%)	18 (15.0%)	NS
Arthralgia	23 (59%)	92 (78.0%)	0.0201
Arthritis	8 (20.5%)	36 (30.5%)	NS
Gastrointestinal tract involvement	22 (56.4%)	63 (53.4%)	NS
Renal involvement SRC	20 (51.3%) 2 (5.1%)	24 (20.4%) 7 (5.9%)	0.0002
Calcinosis	6 (15.4%)	22 (18.6%)	NS
Digital ulcerations	7 (18.0%)	15 (12.7%)	NS
Death	6 (15.4%)	180 (15.2%)	NS
Neoplasm	6 (15.4%)	12 (11.8%)	NS
Overlap syndrome/ATD	6 (15.4%)	21 (17.8%)	NS
Contractures	7 (18.0%)	35 (29.7%)	NS

*ILD* interstitial lung disease, *HRCT* high-resolution computer tomography, *DLCO* diffusing capacity for carbon monoxide, *PAH* pulmonary arterial hypertension, *SRC* scleroderma renal crisis, *ATD* autoimmune thyroid disease

Data were presented as number and percentage. “*P* value of < 0.05 was considered statistically significant”

**Table 3:** Comparison of the serological parameters in group I (late-age onset—after age 60 years) and group II (early-onset before age 60 years) patients with systemic sclerosis

	Group I *n* = 39	Group II *n* = 118	*p*
Anti-topo I (Scl-70)	16 (41%)	50 (42.4%)	NS
Anti-centromere Anti-PM/Scl	12 (31%)	21 (17.8%)	0.07
	2 (5%)	20 (17.9%)	**0.048**
Anti-RNA polymerase III	0 (0%)	19 (16%)	**0.008** NS
Anti-fibrillarin	1 (2.5%)	2 (1.7%)	NS
Anti-Ku	1 (2.5%)	4 (3.4%)	NS
Anti-Th/To	2 (5%)	0 (0%)	**0.014**
Anti-Nor-90	3 (7.7%)	3 (2.5%)	NS
Anti-PDGFR	0	0	NS
anti-Ro-52	9 (23%)	28 (23.3%)	NS

Data were presented as number and percentage. “*P* value of < 0.05 was considered statistically significant”

**Table 4:** Differences between clinical characteristics of systemic sclerosis patients between groups III and IV

	Group III before age 60 years	Group IV after age 60 years	*P*
Number of patients	82	75	NS
dc-SSc	42 (50.6%)	27 (36.0%)	0.0357
lc-SSc	40 (49.4%)	48 (64.0%)	0.654
Gender			
Female	58 (70.7%)	61 (81.3%)	NS
Male	24 (29.3%)	14 (18.7%)	
Age (years)	48.3 ± 10.25 (23–60)	68.65 ± 5.63 (60–81)	NS
Duration of disease (years)	4.17 ± 4.64 (0.0–22.0)	6.27 ± 6.05 (0.0–23.0)	0.000
The time from onset of Raynaud phenomenon to diagnosis (years)	4.74 ± 6.4 (0.0–30.0)	6.27 ± 6.05 (0–23.0)	NS
Age of diagnosis			
Before age 20 years	6 (7%)	0	
20–40 years of age	31 (38%)	4 (5%)	
40–60 years of age	45 (55%)	32 (43%)	
After age 60 years	0	39 (52%)	

Data were presented as number and percentage. “*P* value of < 0.05 was considered statistically significant”

**Table 5:** Comparison of selected clinical parameters in group III (before age 60 years) and group IV (after age 60 years) patients with systemic sclerosis

	Group III *n* = 82	Group IV *n* = 75	*p*
ILD (HRCT)	46 (56.0%)	38 (56.7.3%)	NS
Decreased DLCO	33 (40.24%)	34 (45.3%)	NS
Heart involvement	27 (32.9%)	34 (45.3%)	NS
PAH (ECHO)	12 (14.6%)	19 (25.3%)	0.0923
Myalgia or myositis	11 (13.6%)	11 (14.7%)	NS
Arthralgia	57 (69.5%)	59 (78.7%)	NS
Arthritis	24 (29.3%)	20 (26.7%)	NS
Gastrointestinal tract involvement	38 (46.3%)	47 (62.7%)	**0.0394**
Renal involvement SRC	14 (17.1.36%) 5 (6.0%)	30 (40.0%) 4 (5.3%)	**0.0014**
Calcinosis	17 (21.0%)	11 (14.7%)	NS
Digital ulcerations	12 (14.6%)	10 (14.7%)	NS
Death	16 (19.5%)	8 (10.7%)	0.08
Neoplasm	9 (11.0%)	10 (13.3%)	NS
Overlap syndrome/ATD	11 (13.4%)	16 (21.3%)	NS
Contractures	22 (26.8%)	20 (26.7%)	NS

*ILD* interstitial lung disease, *HRCT* high-resolution computer tomography, *DLCO* diffusing capacity for carbon monoxide, *PAH* pulmonary arterial hypertension, *SRC* scleroderma renal crisis, *ATD* autoimmune thyroid disease

Data were presented as number and percentage. “*P* value of < 0.05 was considered statistically significant”

**Table 6:** Comparison of selected serological parameters in group III (before age 60 years) and group IV (after age 60 years) patients with systemic sclerosis

	Group III *n* = 82	Group IV *n* = 75	*p*
Anti-topo I (Scl-70)	40 (48.8%)	26 (34.7%)	0.080
Anti-centromere Anti-PM/Scl	16 (19.5%)	17 (22.7%)	NS
	14 (17.1%)	8 (10.7%)	NS
anti-RNA Polymerase III	8 (9.7%)	8 (10.7%)	NS
Anti-fibrillarin	2 (2.4%)	1 (1.3%)	NS
Anti-Ku	3 (3.7%)	2 (2.7%)	NS
Anti-Th/To	0	2 (2.7%)	NS
Anti-Nor-90	1 (1.2%)	5 (6.7%)	NS
Anti-PDGFR	0	0	NS
Anti-Ro-52	18 (21.9%)	19 (25.3%)	NS

Data were presented as number and percentage. “*P* value of < 0.05 was considered statistically significant”

## Discussion

In the present study, we have shown marked differences in the clinical and serological spectrum of LAO SSc and EAO SSc, as well as between elder and younger SSc population. It has been shown that the course of systemic sclerosis in the elderly differs significantly from that in younger people. Particularly noteworthy is the fact that involvement of internal organs, heart, kidneys, and pulmonary arterial hypertension, is much more often observed among patients with LAO SSc. Moreover, these groups also differ in their serological profile. We are taking into considerations that differences could also be related to age-related comorbidities.

Some previous clinical studies have explored the effect of age on the disease course with varying conclusions [2]. In one study by Manno et al. [6], 2300 of SSc patients from Johns Hopkins Scleroderma Center were evaluated between 1990 and 2009. The mean age of SSc onset was 45 years, 2084 (91%) of the studied individual had the disease onset prior to the age of 65 years and 216 (9%) over this age. Of those with LAO SSc, 105 (49%) had the onset between 65 and 70 years, 68 (31%) between 70 and 75 years, 36 (17%) between 75 and 80 years, and 7 (3%) after the age of 80. An interesting observation from that study was that patients with LAO SSc had a shorter disease duration and the shorter time to disease diagnosis than those with younger age of SSc onset. Furthermore, Raynaud phenomenon had a less severe course in older patients and the risk of digital ulcers was reduced. However, the LAO SSc patients from the study by Manno et al. were at greater risk of PAH, cardiac disease, and renal impairment in comparison with the EAO SSc group that corresponds well with our findings; but contrastingly to that study, we did not found differences in the presence of muscle weakness between both groups. Additionally, Manno et al. found that patients with LAO SSc had a lower frequency of anti-U1RNP antibodies but a higher prevalence of ACAs compared to EAO SSc [6]. This latter finding is similar to our since we observed a tendency (close to statistical significance) to a higher prevalence of ACAs in the LAO SSc. All these results suggest, coincidentally with our study, that older LAO SSc patients are at higher risk for the significant internal organ morbidity, i.e., pulmonary arterial hypertension, renal impairment, heart disease, and muscle weakness. These findings are consistent with that from the Spanish Cohort [5]. The Spanish Scleroderma Study Group analysed 1037 of SSc patients classified into three groups according to the age of disease onset: age < 30 years (early-onset—195 patients), age between 31 and 59 years (standard onset—651 patients) and age > 60 years (late onset—191 patients). Their findings are in line with the American cohort study showing that that the time to disease diagnosis was shorter in late-onset patients than among younger ones. Furthermore, the LAO SSc patients displayed an increased prevalence of lcSSc with high cardiopulmonary morbidity—manifested as PAH, heart disease, and systemic hypertension. In that Spanish SSc cohort, in accordance with our results, PAH was more common in elderly patients (late onset) than in standard onset group (25% vs. 12%), but late-onset group had also the lowest risk of muscle involvement while in our study there were no statistical differences regarding the frequency of this disease feature between LAO and EAO SSc. Moreover, in the Spanish study, esophageal involvement was the least common in those over 60 years of age, which is also in opposite to our findings as we observed more frequent gastrointestinal involvement in LAO versus EAO SSc. In addition, patients from the elderly group were shown to develop digital ulcers less frequently although they had higher prevalence of ACAs positivity [5]. Interestingly, after adjusting to age and sex, the standardized mortality ratio emerged to be higher in younger SSc patients [5]. Thus, less severe course of the disease may be suggested in LAO SSc group when compared to younger SSc population. Other study was based on EULAR Scleroderma Trials and Research (EUSTAR) database and revealed that of 8554 patients, 123 had an onset of SSc after the age of 75 years [20]. Patients with LAO SSc were also revealed to develop more commonly the limited subtype of SSc and PAH, but fewer patients from this group had digital ulcers. Similarly, elder patients displayed a higher prevalence of ACAs. Regarding SSc-related mortality rate in the EUSTAR cohort, it was higher in the LAO SSc group, even though the survival time from diagnosis was longer compared with younger patients [20]. Apipattarakul et al. analyzed a group of 350 Thai SSc patients and established that 53 (15%) patients had an elderly-onset SSc after the age of 60 years [4], which confirmed previous findings. In contrast to earlier studies, the reported mortality rate was 5.7 times higher in elderly onset SSc, whereas the median survival rate was 11 years shorter than in adult-onset SSc [4]. Achille et al. described the group of 27/246 (11%) patients with SSc diagnosed after the age of 70 and revealed more frequent limited course of the disease and PAH in this group than in the younger SSc patients [21]. Hence, PAH may be the leading clinical sign of the disease at this age. Furthermore, in that study, among the late-onset group, neoplastic disorders were found to occur more commonly than in early-onset patients and this varies from our results—we did not observe the significant differences in the neoplasms’ prevalence according to the age of SSc onset [21]. In the study by Thai authors, which findings of higher prevalence of LAO SSc seems somewhat controversial, the late-onset group included 78 patients (67.8%) from the total of 115 SSc patients. However, in that study, the age limit for the classification of late-onset SSc was determined as > 50 years. Moreover, the authors reported that digital pitting scars, xerophthalmia, joint contractures, and systemic hypertension were more prevalent in LAO SSc than EAO SSc, whereas they did not find significant differences regarding the incidence rate of cardiopulmonary involvement between the two subgroups [22], which all is at odds with results of our study *p*. These discrepancies, including higher frequency of LAO and a different clinical spectrum of the LAO SSc may result either from lower age limit for late-onset SSc in the Thai study or population bias. In fact, according to the largest worldwide systemic sclerosis database, EUSTAR, cardiovascular involvement in older SSc patients is reported to be higher. Importantly, older patients are at an elevated risk of more severe disease course and greater degree cardiac involvement at early stages of SSc, although the diffuse SSc subset with a presence of anti-Scl-70 antibodies was more frequent in the younger subgroup [23, 24]. The results of our study are in line with findings of most of previous works. About 25% of SSc patients in our study group had the onset of the disease after 60 years old. Our findings also confirm that patients with LAO SSc are at greater risk of heart disease, particularly pulmonary arterial hypertension, when compared to younger patients. Thereby, PAH may be of a significant concern among older SSc patients. However, it remains unclear whether the higher frequency of primary heart involvement in the late-onset was due to SSc or should be linked to the normal ageing process and comorbidities. We also shown a renal involvement (but other than SRC) to be a frequent condition among late-onset SSc group, which is in contrast to earlier findings by Alba et al. who did not report increased renal involvement rates in patients with late-onset SSc [5]. A possible explanation for this discrepancy may be that the authors of the Spanish study considered only patients with SRC, whereas in our work patients with an elevation of creatinine beyond 1.3 mg/dL or > 2 + protein in urine dipstick were considered. Moreover, in our study, the deterioration of kidney function in the LAO SSc group may also be associated with age-related impairment of circulation, comorbidities, and their pharmacotherapy. Noteworthy, we have previously demonstrated that there is a relationship between kidney involvement and the positivity for some antiphospholipid antibodies (aPL) in patients with SSc [25]. Yao et al. revealed that the incidence of antiphospholipid syndrome (APS) increases with age especially in the female population, and rises rapidly after the age 60 years old [26].

Consistent with the literature data, our study found that the incidence of dcSSc is lower in patients over 60 years old compared to those before 60, but regardless of the age of the disease onset [20, 23]. In most studies, the limited subtype of the disease and the presence of ACAs predominate in the LAO SSc group [5, 20]. In our observations, the late-onset patient group exhibited a tendency to higher prevalence of ACAs and significantly lower levels of either anti-RNA polymerase III antibodies or anti-Pm/Scl antibodies. The lower frequency of anti-RNA polymerase III antibodies in the LAO SSc group could be an outcome of a lower prevalence of dcSSc, since these autoantibodies are known to be a good marker of diffuse subtype of SSc [9]. Scalapino et al. describe that the phenotypic variation by age has been established in younger SSc patients (juvenile-onset SSc) who reported an increased prevalence of overlap syndrome with myositis, SSc-specific antibodies (anti-PM/Scl antibodies), and improved survival compared with adult-onset SSc [27].

In conclusions, we have demonstrated that the clinical and serological spectrum of late-onset SSc is markedly different from that of early-onset disease and patients with LAO SSc exhibit an increased risk of specific organ involvement, primarily pulmonary arterial hypertension and renal involvement. This is mostly consistent with the results of the majority of similar studies and confirm that age at the disease onset may be an important factor influencing phenotypic variations in the clinical picture and outcomes of patients with SSc [5, 6, 20, 28, 29]. Determining these various characteristics and the awareness of the risk of certain organ manifestations in LAO SSc, particularly pulmonary arterial hypertension, should result in special care for these patients and may help to improve the personalized management of the disease; however, the differences between younger age versus older age onset SSc patients are yet to be explained and thus require further studies. The poor prognosis or higher mortality rates among LAO SSc patients, both of which are recorded in the majority of studies might be related to comorbidities and an increased risk of neoplasms in older SSc patient population [30–33].

Although our study has successfully demonstrated the different course of SSc in early and late-onset patients, it has a limitation of not reporting a control group of non-SSc age-matched individuals. Thus, the study will be continued with age-matched healthy cohorts.
