# Correction: Late-age onset systemic sclerosis—clinical and serological characteristics

**DOI:** 10.1007/s10067-024-07090-4

**Published:** 2024-08-16

**Authors:** Ewa Wielosz, Katarzyna Wiąk-Walerowicz, Ewa Łyś, Aleksandra Lipska, Magdalena Dryglewska, Maria Majdan

**Affiliations:** Department of Rheumatology and Connective Tissue Diseases, Medical University of Lublin, Jaczewskiego 8 St., 20-090 Lublin, Poland

**Correction: Clinical Rheumatology (2024) 43:2565-2572**

10.1007/s10067-024-07025-z

In this article the author name 'Maria Majdan 'was incorrectly captured as corresponding author. The correct corresponding author should be 'Ewa Wielosz'.

The original article has been corrected.

